# Modeling Initiation of Ewing Sarcoma in Human Neural Crest Cells

**DOI:** 10.1371/journal.pone.0019305

**Published:** 2011-04-29

**Authors:** Cornelia von Levetzow, Xiaohua Jiang, Ynnez Gwye, Gregor von Levetzow, Long Hung, Aaron Cooper, Jessie Hao-Ru Hsu, Elizabeth R. Lawlor

**Affiliations:** 1 Department of Pediatrics, Children's Hospital Los Angeles, Los Angeles, California, United States of America; 2 Departments of Pediatrics and Pathology, University of Michigan, Ann Arbor, Michigan, United States of America; Institute of Genetics and Molecular and Cellular Biology, France

## Abstract

Ewing sarcoma family tumors (ESFT) are aggressive bone and soft tissue tumors that express EWS-ETS fusion genes as driver mutations. Although the histogenesis of ESFT is controversial, mesenchymal (MSC) and/or neural crest (NCSC) stem cells have been implicated as cells of origin. For the current study we evaluated the consequences of EWS-FLI1 expression in human embryonic stem cell-derived NCSC (hNCSC). Ectopic expression of EWS-FLI1 in undifferentiated hNCSC and their neuro-mesenchymal stem cell (hNC-MSC) progeny was readily tolerated and led to altered expression of both well established as well as novel EWS-FLI1 target genes. Importantly, whole genome expression profiling studies revealed that the molecular signature of established ESFT is more similar to hNCSC than any other normal tissue, including MSC, indicating that maintenance or reactivation of the NCSC program is a feature of ESFT pathogenesis. Consistent with this hypothesis, EWS-FLI1 induced hNCSC genes as well as the polycomb proteins BMI-1 and EZH2 in hNC-MSC. In addition, up-regulation of BMI-1 was associated with avoidance of cellular senescence and reversible silencing of p16. Together these studies confirm that, unlike terminally differentiated cells but consistent with bone marrow-derived MSC, NCSC tolerate expression of EWS-FLI1 and ectopic expression of the oncogene initiates transition to an ESFT-like state. In addition, to our knowledge this is the first demonstration that EWS-FLI1-mediated induction of BMI-1 and epigenetic silencing of p16 might be critical early initiating events in ESFT tumorigenesis.

## Introduction

Ewing's sarcoma family tumors (ESFT) are aggressive bone and soft tissue tumors that primarily affect children and young adults [1]. They are largely undifferentiated tumors that are genetically characterized by expression of fusion oncogenes resulting from chromosomal translocations involving *EWSR1* (*EWS*), (or rarely *TLS*), and *FLI1* or another member of the ETS family of transcription factors [2]. Despite its action as an oncogene in ESFT, EWS-FLI1 is toxic to most cells, inducing arrest and death [3], [4]. Although experimental inactivation of p53 and p16 in primary cells can induce tolerance to EWS-FLI1, genetic mutations in these tumor suppressors are present in only a minority of ESFT [5]. Thus, alternate mechanisms of tumor suppressor gene inactivation may exist in the ESFT cell of origin.

The cellular origin of ESFT remains both elusive and controversial. Recent studies have shown that bone marrow-derived human mesenchymal stem cells (BM-MSC) are permissive for EWS-FLI1 and that ectopic expression of EWS-FLI1 in these cells initiates transition to an ESFT-like cellular phenotype [6], [7]. However, the immature neural phenotype of many tumors, along with their gene expression signatures and their disposition to neural differentiation also implicate a neural crest origin [8], [9], [10], [11]. Like ESFT cells, neural crest stem cells (NCSC) are highly migratory and invasive and during embryogenesis travel to numerous tissues throughout the body [12]. Significantly, studies in model organisms have additionally shown that some MSC are derived from the neural crest [13], [14]. Thus, this partially shared ontogeny leads to the unifying hypothesis that ESFT might arise from malignant transformation of MSC of either mesodermal or neural crest origin [15].

Studies in chick embyros have demonstrated that expression of EWS-FLI1 disrupts normal NCSC development [16]. However, studies with human neural crest cells have not yet been reported and have been challenged by the very transient nature of the neural crest in early embryogenesis and the rarity of NCSC in post-natal tissues. We recently established an efficient method to generate NCSC from *in vitro* differentiating human embryonic stem cells (hESC) [17]. These cells display the genetic, phenotypic and functional characteristics of NCSC and differentiate *in vitro* and *in vivo* into neural crest derivatives [17]. For the current study we used this model to study the consequences of EWS-FLI1 expression in human NCSC and their progeny.

## Materials and Methods

### Ethics statement

All human tumor specimens were obtained in compliance with HIPAA regulations and following protocol review by the Committee for Clinical Investigation at Children's Hospital Los Angeles. Samples were provided to investigators as anonymized RNA with no links to patient identifiers. The study (05-545) was reviewed in an expedited manner on 2/12/2007 and was approved and deemed to meet the criteria for non-human subjects research and for a waiver of authorization/informed consent. All animal studies were performed following full protocol review and approval by the Institutional Animal Care and Usage Committee (IACUC) of Children's Hospital Los Angeles (protocol 216-07).

### Cell culture

WA-09 hESC were purchased from Wicell (Madison, WI) and hESC-derived neural crest stem cells (hNCSC) generated as described [17]. FACS-sorted p75+ hNCSC cells were maintained in self-renewal medium (DMEM-F12 (1∶1) N2 and B27, 20 ng/ml bFGF, 20 ng/ml EGF, 20 nM IGF-1 (all from Gibco), 0.1 mM β-mercaptoethanol) on 6-well ultra-low attachment plates (Corning, Lowell, MA) at a density of 5×10^3^ cells/ml. To promote generation of MSC-like cells, hNCSC were plated at a cell density of 10–20×10^3^ cells/cm^2^ in self-renewal media on 6-well plates that were pre-coated with 15 µg/ml Polyornithine (Sigma), 1 µg/ml laminin (Millipore) and 10 µg/ml fibronectin (Invitrogen). Three unique human bone marrow-derived MSC (BM-MSC) lines were obtained from Dr. D. Prockop (Tulane University) and maintained at low density in αMEM with 10% FBS (Invitrogen), L-Glutamine, NEM Nonessential Amino Acids and Sodium Pyruvate (all from Cellgro). All studies were performed with the approval of Institutional Human Pluripotent Stem Cell Research Oversight Committees.

### Differentiation assays

For non-specific neural crest differentiation of hNCSC, self-renewal media was changed to DMEM/F12 (1∶1) supplemented with N2, B27 and 5% Hyclone® fetal bovine serum (FBS) (Thermo scientific). For adipogenic differentiation, media of confluent cells was changed to αMEM (Cellgro) containing 10% FBS, 10 µg/ml insulin (Sigma), 10 µM Dexamethasone (Sigma) and 0.5 mM IsoButylMethylXanthine (Sigma). For osteogenic differentiation, media was changed to Minimum Essential Medium, Alpha 1× (Cellgro) containing 10% FBS, 10 mM β-glycerolphosphate (Sigma), 0.1 µM Dexamethasone (Sigma) and 200 µM L-ascorbic acid (Sigma). Defined media were changed every 3 days and cells fixed in 4% paraformaldehyde after 21 days. Terminal adipocytic and osteoblastic differentiation was assessed by staining fixed cells with 0.5% Oil Red-O (Sigma) and 1% Alizarin Red S (Sigma), respectively.

### Immunostaining and Western blot

Cells were grown in pre-coated chamber slides (LAB-TEK, Nun International), fixed in 4% paraformaldehyde and permeabilized with 0.1% Triton X-100. After rinsing in PBS the slides were incubated for one hour in blocking solution (1% BSA and 5% donkey serum in PBS). Blocked slides were incubated with primary antibodies for 1 hr at 37°C, washed in PBS, incubated with fluorescent-labeled secondary antibodies for 1 hr and then visualized with a Lexica DM RXA Upright Fluorescence Microscope (Applied Spectral Imaging, Inc., Carlsbad, CA). Primary antibodies used were: V5 (1∶500, Invitrogen), GFP (1∶1000, Abeam, Cambridge, MA). Secondary antibodies were: Alexi fluorophore 488- conjugated Donkey-Anti Rabbit (1∶1000, Molecular Probes- Invitrogen) and Cy3-conjugated Goat Anti-Mouse (1∶500; Jackson Immune Research Laboratories). In some samples, nuclei were counterstained with 4′, 6-diamidino-2- phenylindole (DAPI). Western blots were performed using standard procedures and the following antibodies at 1∶1000 dilutions: V5 (Invitrogen), BMI-1 (Millipore); EZH2, GAPDH, Cyclin D1 (Cell Signaling Technology); p16, Actin, (Santa Cruz Biotechnology); p21 (Abcam); p53 (1∶2000, Cell signaling).

### Flow cytometry and cell growth assays

Cells were dissociated with Accutase (Millipore) and washed with L15 medium with 10 mM HEPES and 1 mg/ml BSA. Resuspended cells were blocked with anti-human Fc-receptor (Miltenyi Biotec, Germany) and incubated with antibodies for 10 min at 4°C in the dark (phycoerythrin (PE)-conjugated anti-p75, PE-CD29, PE-CD73, Allophycocyanin (APC)-conjugated CD44 (Miltenyi Biotec, Germany), PE-CD105 (Santa Cruz Biotechnology), APC-CD90 (BD Biosciences Pharmingen) and PE Mouse IgG2aκ-, PE Mouse IgG2b-, PE Mouse IgG1κ-, APC Mouse IgG1κ-isotope controls (all BD Biosciences Pharmingen). For the p75 and CD73 double staining cells were incubated with PE-p75 and CD73 (BD Pharmingen) antibodies followed by a FITC-conjugated goat anti mouse antibody (Jackson ImmunoResearch, West grove, PA). Cells were washed with staining media and then analyzed using a FACS Caliber instrument (BD Biosciences, San Jose, CA). Gates were defined relative to untransduced and isotope-control cells and FCS Express software package (De Novo Software) used for data analysis. To isolate GFP positive populations, dissociated cells were sorted on a FACS Vantage or FACS Aria (BD Biosciences). Cell cycle analysis was performed on a FACScan instrument (Becton Dickinson) and data analyzed using Flow-Jo (Tree Star, Ashland, OR). Beta-galactosidase staining for cellular senescence was performed as previously described [18]. To assess p53 function cells were exposed to 1500 Rad gamma irradiation and whole cell lysates collected after 24 hrs.

### EWS-FLI1 expression construct and BMI-1 knockdown vector

The coding sequence of EWS-FLI1, minus its stop codon, was PCR amplified from pcDNA/TO-EF (kindly provided by Dr. T. Triche) and inserted into pENTR/D-TOPO (Invitrogen, Carlsbad, CA). EWS-FLI1 was then cloned in-frame into pLenti4/TO/V5-DEST (Invitrogen) by LR recombination according to the manufacturer's instructions. EWS-FLI1-V5 was PCR-amplified from the pLenti4/TO/V5-DEST vector, following site-directed mutagenesis of the internal EcoRI site (conserving amino acid sequence), and cloned into the NheI and EcoRI restriction sites of the pCLS backbone to generate pCLS-EF. The pCLS lentiviral vector is a derivative of pCL1 [19], modified to include a foot-and-mouth-disease virus (FMDV)-derived 2A self-cleaving peptide sequence in place of the internal ribosomal entry site upstream of the EGFP reporter gene [20]. Furin cleavage sites (R-A-K-R) were inserted between EWS-FLI1 and the 2A sequence in order to achieve full cleavage of the translated EWS-FLI1 protein from residual 2A peptide [21], [22]. Empty vector with 2A-EGFP alone served as a control (pCLS-CV).

For the BMI-1 knock down experiments, the pCLS lentiviral backbone was modified as follows: First, the rtTA IRES Puro expression cassette was amplified from pTRIPZ (Open Biosystems, Huntsville, AL) using the following primers: 5′ TCACTCGGCGCGCCGCCACCATGTCTAGGCTGGACAA 3′ and 5′ TTTACTTGTACATCAGGCACCGGGCTTGCGG 3′. The resulting PCR product was then cut with AscI and BsrGI (both New England Biolabs, Ipswich, MA) and ligated into pCLS. Two additional restriction enzyme sites (AgeI and NheI) were inserted into the vector upstream of the SFFV U3 promoter by site directed mutagenesis using 5′-phosphorylated oligonucleotides (fw: 5-GGATCCACCGGTCGCCACCATGAGCGA-3′ and rev: 5′-GTTTAAACGCGATCTGACGGTTCACTAAACGAG-3′). Finally, the expression cassette containing the TRE/CMV minimal promoter, tRFP and the shRNA cassette was amplified from pTRIPZ using the following primers: 5′-CCCGGGTCCGGACTATGCCGATGATTAATTGTCAACACGT-3′ and 5′-GCGTTAGCTAGCGGCCGGCCGCATTAGTCTTCCA-3′. The resulting PCR product was cut with BspEI and NheI and ligated into AgeI and NheI sites in the modified pCLS vector to generate an shRNA-cassette-ready TRE/tRFP/rtTA/IRES/Puro backbone (pCLSTRRIP). To selectively target BMI-1 for knockdown, a miR-30-based shRNA expression cassette was cloned by primer-extension PCR using the following primers: miR30fw: 5′-CAGAAGGCTCGAGAAGGTATATTGCTGTTGACAGTGAGCG 3′, miR30 rev: 5′-CTAAAGTAGCCCCTTGAATTCCGAGGCAGTAGGCA-3′ and BMI1-shRNA: 5′-TGCTGTTGACAGTGAGCGAAGCGGTAACCACCAATCTTCTTAGTGAAGCCACAGATGTAAGAAGATTGGTGGTTACCGCTGTGCCTACTGCCTCGGA-3′ (BMI-1-targeted sequence, underlined, is flanked by miR-30 sequence). The resulting PCR product was cut with XhoI and EcoRI and ligated into pCLSTRRIP. All PCRs were carried out using Phusion High Fidelity Polymerase (New England Biolabs, Ipswich, MA) and the resulting vectors were sequenced verified. Inert non-silencing shRNA sequence was excised from control pTRIPZ vector (Open Biosystems, Huntsville, AL) and cloned into pCLSTRRIP using EcoRI and XhoI restriction sites.To activate BMI-1 knockdown 100 ng/ml doxycyline was added to the culture media of stably infected cells.

### Lentiviral transductions

To generate viral supernatant, 293FT cells were transfected with pCLS-CV or pCLS-EF, the packaging plasmid pCD/NL-BH*DDD (Addgene plasmid 17531) and pMD2.G (VSV-G; Addgene plasmid 12259) using Polyethylenimine (1 mg/ml, Sigma-Aldrich). The day after transfection, cells were cultivated for 6–8 hours with sodium butyrate (10 mM) and viral supernatant collected after 48 hrs. Freshly isolated hNCSC were transduced with concentrated EWS-FLI1 or EGFP virus at a multiplicity of infection of 2. Transduction efficiencies were reproducibly around 80%. Infected cells were isolated by FACS-sorting for EGFP expression. For BMI-1 knockdown studies 293T cells were transfected with packaging plasmids as above along with inducible shBMI1 or shNS constructs. Viral supernatant was collected and EF-NC cells were transduced with shBMI1 or shNS virus and then selected in puromycin (2 ug/ml) for 24 hrs prior to treatment with doxycyline. Cells were cultured in puromycin and doxycyline for one week.

### Gene expression studies

Total RNA was isolated using the miRNeasy kit with in-column DNAse I treatment (Qiagen). cDNA generated with iScript (Bio-Rad, Hercules, CA) was amplified by PCR using standard conditions (Primer information provided in Table S4). Quantitative reverse transcriptase PCR (RT-PCR) was performed using validated TaqMan Assays (Applied Biosystems) on an Applied Biosystems 7900HT PCR system and expression normalized relative to GAPDH as described [23]. For whole genome expression assays, total RNA was processed and hybridized to Affymetrix HuEx 1.0 arrays in the CHLA/USC Genome Core according to Affymetrix protocols. RNA from primary tumors was obtained from Children's Oncology Group and CHLA tumor banks. HuEx cel files from normal human tissues were downloaded from the Affymetrix Netaffx website (www.netaffx.com). Cell line data were kindly provided by Dr. T. Triche. Normalization and statistical analysis of microarray data were performed as detailed below. Gene expression data are available in the GEO repository (GSE21511).

### Affymetrix HuEx data analysis

Data for core probeset regions were quantile normalized using robust multi-chip averaging in the Partek Genomics Suite software platform (Partek, St. Louis, Mo). Transcript level data were derived from normalized exon data using median summarization and all further analyses performed on transcript-summarized data. To identify EWS-FLI1 targets, data from control vector-transduced and EWS-FLI1-transduced samples from 3 independent experiments were normalized as above and EWS-FLI1 targets identified by 2-way ANOVA, controlling for the effects of experimental batch. Analysis of gene ontology was performed using DAVID bioinformatics resources [24].

## Results

### hNCSC possess mesenchymal differentiation potential

**Figure 1:**
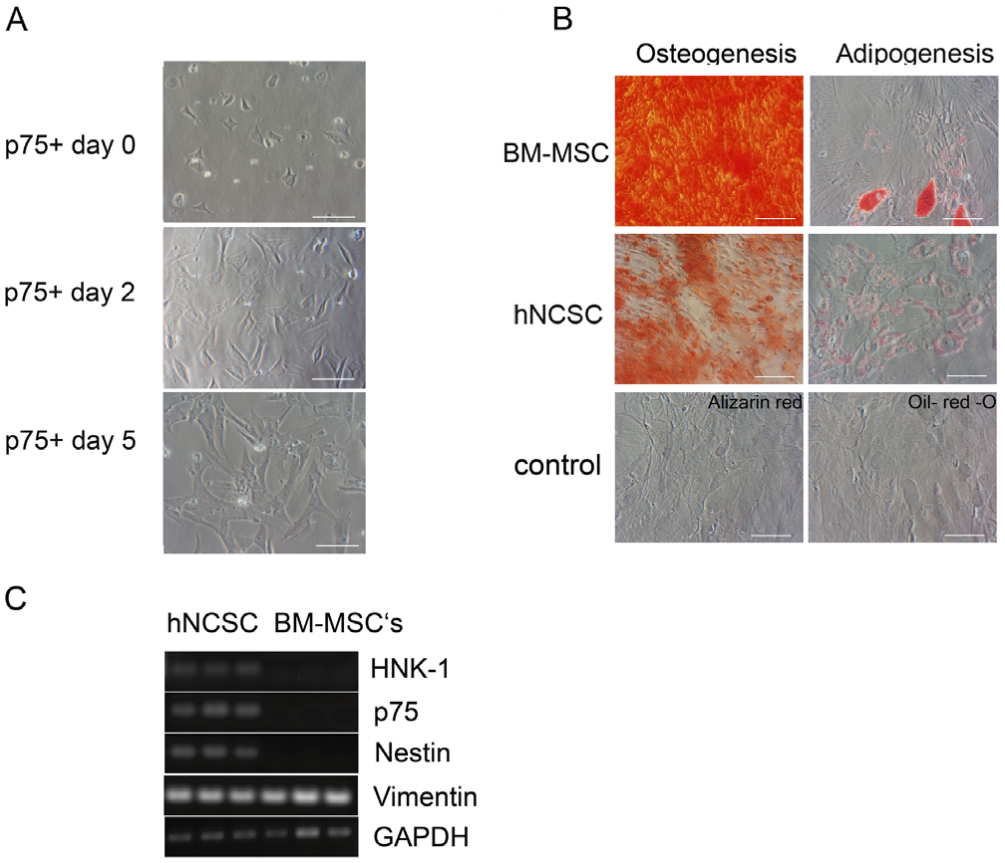
hNCSC have mesenchymal differentiation potential. (**A**) Within 2 days of transfer to adherent plates p75+ hNCSC initiated a change from small, NCSC-like to larger MSC-like cells. By 5 days all cells were mesenchymal in appearance (In all images scale bars = 100 µm). (**B**) Oil-Red O- and Alizarin Red staining after 3 weeks in defined media confirmed that hNCSC can be induced to differentiate into adipocytes and osteoblasts, respectively, albeit less robustly than BM-MSC. Simliarly stained BM-MSC that had been exposed to standard growth media are shown as negative controls. (**C**) RT-PCR showed that hNCSC express the mesenchymal marker *VIM (vimentin)* as well as NCSC genes *B3GAT1 (HNK-1)*, *NGFR (p75)*, and *NES (nestin)*. In contrast, BM-MSC (3 different lines as detailed in methods) do not express NCSC genes. BM-MSC: bone marrow-derived MSC; hNCSC: hESC-derived NCSC.

**Figure 2:**
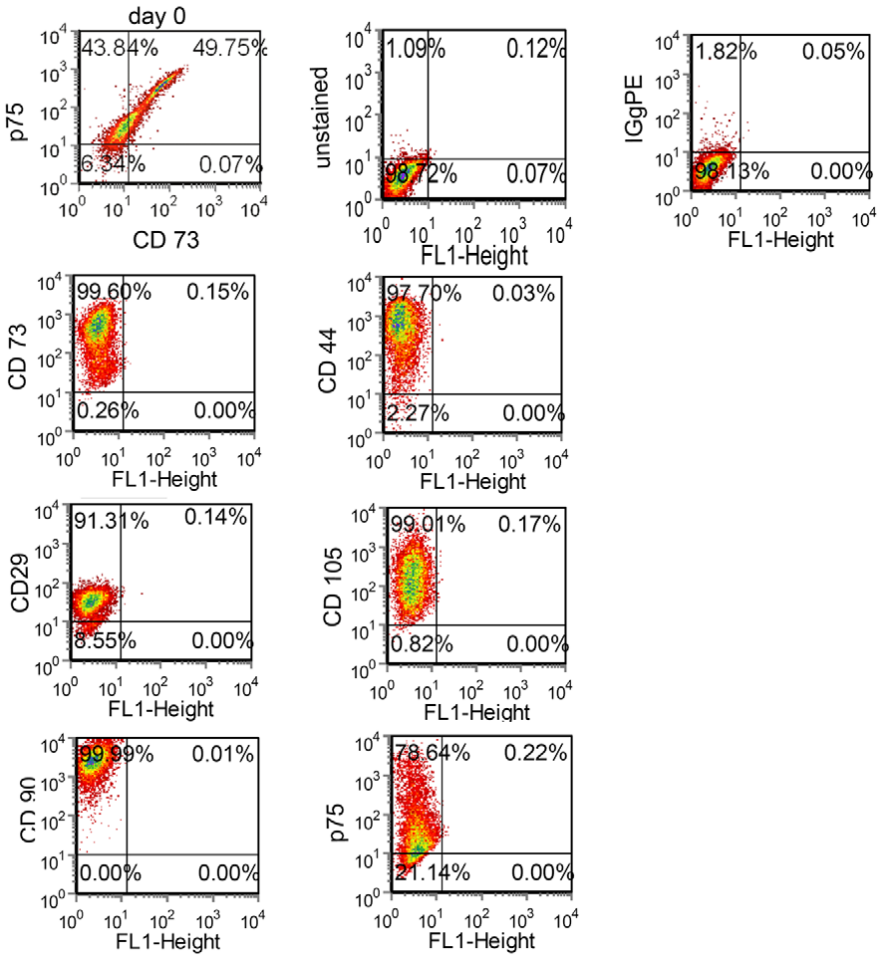
Flow cytometry confirms MSC markers in adherent hNCSC. hNCSC were isolated from *in vitro* differentiating hESC using p75-FACS. Nearly 50% of the cells also expressed the MSC marker CD73 on Day 0. After 2 days in adherent conditions the morphology of isolated cells changed from small cuboidal cells to larger mesenchymal cells (see Fig. 1A). Consistent with this morphologic change flow cytometric analysis shows that after 5 days in culture most cells continue to express p75 but, in addition, nearly all cells express the MSC-associated markers CD73, CD105, CD90, CD29, and CD44.

Developmental models have recently established that small numbers of MSC are derived from the neural crest [13], [14], [25]. Given the neural crest phenotype and putative MSC origin of ESFT, these cells are attractive candidate cells of origin. We have previously shown that multipotent, self-renewing hNCSC can be isolated from hESC and that these cells can be differentiated *in vitro* and *in vivo* into neural crest progeny [17]. To more fully define their mesenchymal potential hNCSC were generated as previously described [7], [17], [26] and then cultured in adherent conditions on poly-l-ornithine, laminin and fibronectin coated plates. After 2 days in adherent conditions small cuboidal hNCSC were observed to adopt a larger more mesenchymal morphology (Fig. 1A) and exposure of these cells to defined media induced differentiation into adipogenic and osteogenic progeny (Fig. 1B). Importantly, however, despite rapid adoption of a phenotype reminiscent of BM-MSC, freshly isolated hNCSC cells were distinct from BM-MSC in their expression of NCSC-associated genes. (see Fig. 1C and discussion of microarray data below). Flow cytometric analyses confirmed that after several days in adherent culture these hNCSC-derived MSC (hereafter termed hNC-MSC) expressed high levels of both p75 as well as MSC markers CD73, CD44, CD29, CD105 and CD90 (Fig. 2). In addition, hNC-MSC retained the ability to form peripherin-positive neurons thus demonstrating their multi-potent neuro-mesenchymal properties.

### Undifferentiated hNCSC and hNC-MSC are permissive for EWS-FLI1

**Figure 3:**
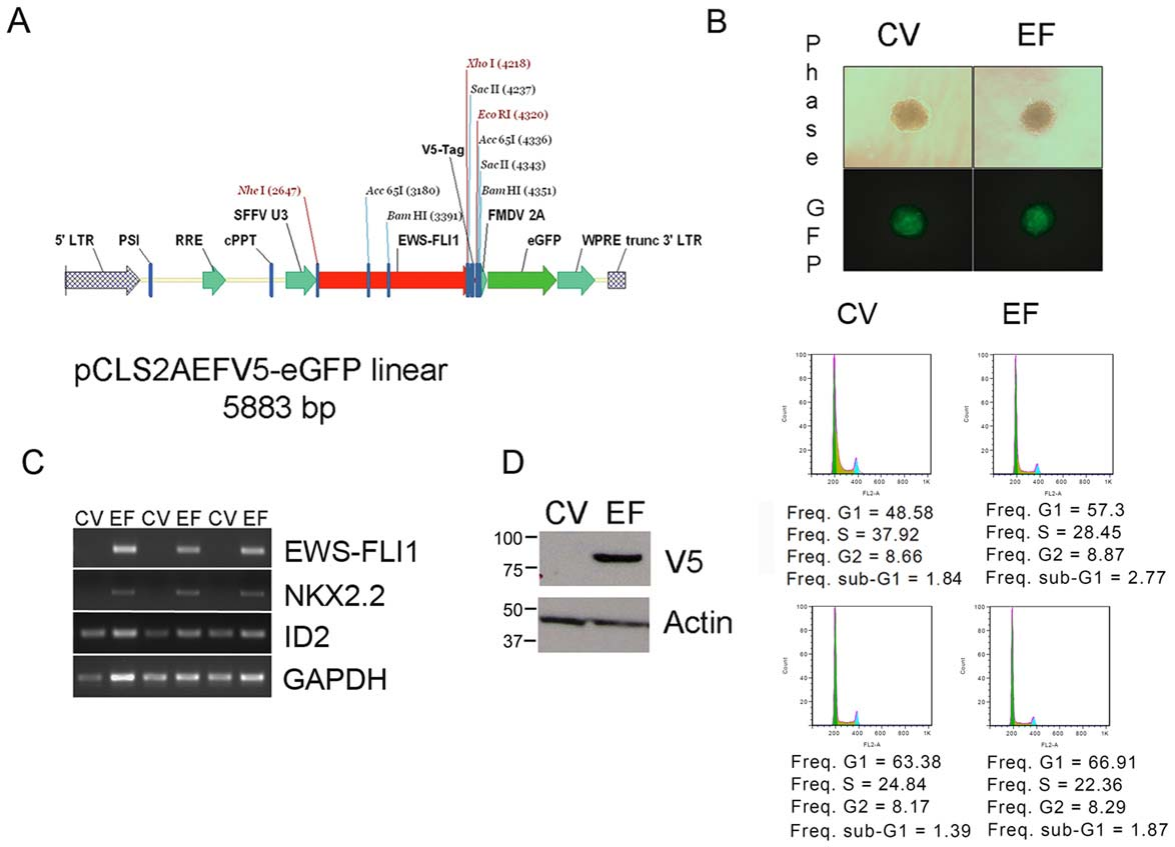
hNCSC and hNC-MSC tolerate expression of EWS-FLI1. (**A**) pCLS-EWS-FLI1-V5-2A-EGFP lentiviral expression vector. (**B**) hNCSC transduced with either control (CV)- or EWS-FLI1-vectors (EF) formed neurospheres in non-adherent culture (phase contrast (top) and GFP-filters (bottom) 100×). (**C**) RT-PCR confirmed expression of *EWS-FLI1* in EF cells and also induction of known EWS-FLI1 target genes *NKX2.2* and *ID2*. (Results are shown for 3 independent experiments). (**D**) Cell cycle was assessed in adherent EGFP+ hNC-MSC 6 weeks after lentiviral transduction. Western blot (left) confirmed continued robust expression of the EWS-FLI1-V5 protein and flow cytometric analysis of DNA content (right) showed continued proliferation and minimal cell death in both EF and CV cells in 2 independent experiments.

To establish whether hNCSC are permissive for EWS-FLI1, freshly sorted p75+ cells were transduced using an EWS-FLI1 lentiviral vector (Fig. 3A) and transferred to neurosphere conditions within 24 hrs. Both EWS-FLI1 (EF-NC) and control vector-transduced (CV-NC) cells rapidly generated EGFP+ spheres that continued to grow in size confirming both successful transduction and retention of the ability to proliferate as neurospheres (Fig. 3B). RT-PCR confirmed expression of *EWS-FLI1* as well as induction of known downstream target genes *NKX2-2* and *ID2* (Fig. 3C). Similarly, when transferred to adherent conditions and passaged as hNC-MSC, cells continued to expand and no substantial differences in cell proliferation or death were observed between control and EWS-FLI1+ cells (Fig. 3D).

### EWS-FLI1 modulates expression of previously characterized as well as novel target genes in hNC-MSC

To characterize the effects of EWS-FLI1 on gene expression in hNC-MSC, transduced cells were expanded in self-renewal media for 5 days in adherent conditions and then profiled using Affymetrix arrays. Over 800 transcripts were significantly modulated by EWS-FLI1 (FDR<0.1 and fold change >1.5; Table S1A). Comparison of EWS-FLI1 targets in hNC-MSC to previously published studies of EWS-FLI1-transduced BM-MSC [7], [26] revealed moderate overlap with pediatric BM-MSC but only minimal overlap with adult BM-MSC (306 *vs.* 154 genes in common) (Table S1B). Overlap between EWS-FLI1 target genes in hNC-MSC and ESFT cell lines [27] was also minimal with only 68 genes being commonly regulated in both model systems (Table S1B). This analysis reaffirms the cell-context dependency of many EWS-FLI1 targets but also identifies a core set of genes that are regulated by EWS-FLI1 irrespective of cell type (Table 1).

**Table 1:** Core target genes significantly regulated by EWS-FLI1 in BM-MSC*, ESFT cell lines**, and hNC-MSC.

Gene Symbol	EWS-FLI1 Induced or Repressed	Gene Symbol	EWS-FLI1 Induced or Repressed
ACTN2	IND	MAP2K6	IND
AKAP7	IND	MAPT	IND
ALG6	IND	MKKS	IND
ATP1A1	IND	NELL2	IND
BCL11B	IND	NKX2-2	IND
C13orf18	IND	PCSK2	IND
CADPS2	IND	PFTK1	IND
CD83	IND	PPP1R1A	IND
CSPG5	IND	PTGER3	IND
DAPK1	IND	RCOR1	IND
DCLRE1B	IND	RHOH	IND
DNAJC12	IND	SH2B3	IND
EFNB1	IND	SLC24A3	IND
EPB41	IND	SLC5A6	IND
EZH2	IND	SORD	IND
FCGRT	IND	STEAP3	IND
FOS	IND	SYNE2	IND
GLG1	IND	TRHDE	IND
GNA14	IND	UTS2	IND
GRK5	IND	WWOX	IND
HOOK1	IND	CDC42EP3	REP
IL1RAP	IND	IL6ST	REP
ITGB2	IND	LOC26010	REP
KDSR	IND	MID1	REP
LBH	IND	PALLD	REP
LDB2	IND	PRSS23	REP

*Riggi et al. 2010,

**Kauer et al. 2008.

### ESFT are genetically similar to hNCSC and EWS-FLI1 initiates reprogramming of hNC-MSC to a more primitive NCSC state

**Figure 4:**
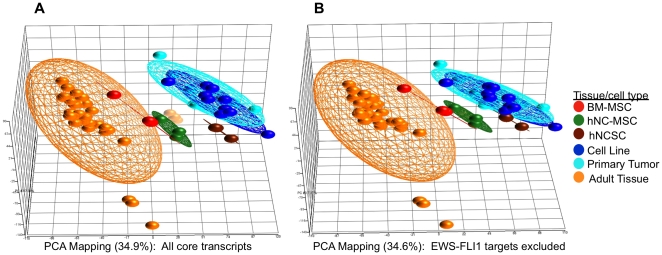
Whole genome expression profiling confirms that ESFT cell lines and tumors are most closely related to hNCSC. (**A**) Unsupervised 3D principal components analysis (PCA) of freshly isolated hNCSC (N = 3), hNC-MSC (N = 3), adult bone marrow-derived MSC (BM-MSC; N = 3), 10 primary ESFT, 10 ESFT cell lines and 11 adult tissues (each in triplicate) shows that ESFT cluster more closely with stem cells than any adult tissue and are most similar to undifferentiated hNCSC. Notably testes and cerebellar tissues cluster separately from other adult tissues (breast, heart, kidney, liver, muscle, pancreas, prostate, spleen, thyroid). All 17,881 core transcripts represented on the array were included for this analysis. (**B**) Unsupervised clustering of samples in (A) was repeated after exclusion of 1,676 EWS-FLI1 target genes. ESFT continue to cluster with hNCSC.

Malignant tumors often share genetic and phenotypic characteristics of the normal cells from which they arose. In attempt to define the cell of origin of ESFT prior studies have used gene expression profiling to compare established tumors to different cell and tissue types [7], [11], [27], [28]. Although these studies collectively favor either an MSC or neural crest stem or progenitor cell of origin, to date no direct comparison between ESFT and NCSC has been reported. We therefore performed unsupervised clustering of normalized expression array data from 10 primary ESFT, 10 ESFT cell lines, hNCSC, hNC-MSC, BM-MSC and 11 different normal adult tissues. As shown, consistent with earlier studies, the gene expression signature of ESFT was found to be more similar to stem cells than to differentiated tissues (Fig. 4A). Additionally, at a whole genome level, ESFT were more related to hNCSC than to BM-MSC. To address whether histogenesis or EWS-FLI1 effects are primarily responsible for the NCSC-like state of ESFT cells we repeated unsupervised clustering of the aforementioned samples after excluding published [26], [27], [29] as well as novel (from the current study) EWS-FLI1 target genes. As shown, even in the absence of 1,676 EWS-FLI1-modulated transcripts, ESFT clustered with stem cells and retained close identity with hNCSC (Fig. 4B).

EWS-FLI1 can partially activate a NCSC signature and trigger an ESFT initiation program in heterologous cells [7], [26], [30]. To determine if EWS-FLI1 initiated reactivation of the hNCSC program in hNC-MSC, we first defined differences between these two cell populations. Consistent with phenotypic observations (Fig. 1 & 2), the molecular signature of hNC-MSC after 5 days in adherent culture was fundamentally altered compared to undifferentiated hNCSC. In particular, hNC-MSC showed significant up-regulation of mesenchymal gene expression and down-regulation of neuroectodermal genes and at a whole genome level these cells clustered more closely with BM-MSC than their hNCSC ancestors (Tables S2A & S2B and Fig. 4). Importantly, however, after only 5 days EWS-FLI1 reactivated expression of 81 hNCSC-associated genes and inhibited expression of 126 hNC-MSC-associated genes (Table S3A). Gene ontologic analysis of these EWS-FLI1-modulated targets confirms significant reactivation of neuroectodermal and concomitant repression of mesodermal differentiation programs in EWS-FLI1 expressing cells (Table S3B).

Together these gene expression profiling studies suggest that maintenance of the NCSC genetic program is central to ESFT pathogenesis and that both the cell of origin and expression of EWS-FLI1 contribute to this state.

### EWS-FLI1 expression in hNCSC induces polycomb proteins and represses p16

**Figure 5:**
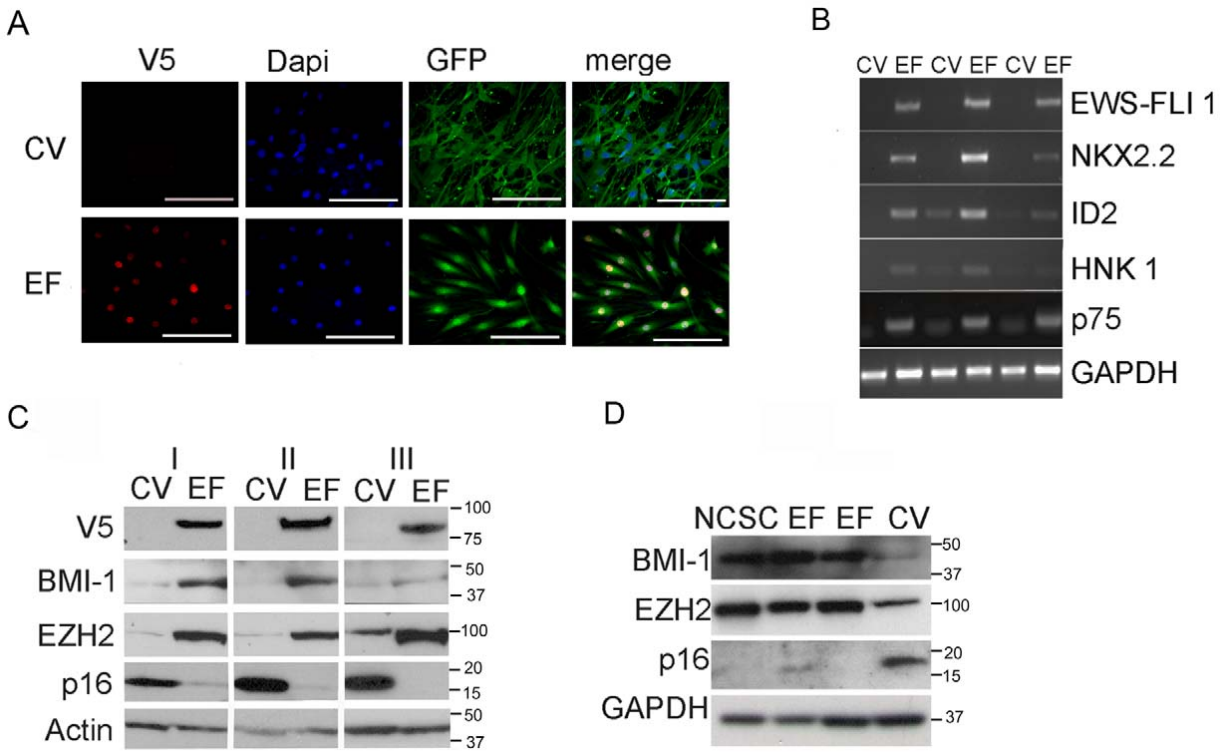
EWS-FLI1 induces a NCSC phenotype in hNC-MSC. (**A**) EWS-FLI1 (EF-NC) and control vector (CV-NC) transduced hNC-MSC were passaged in serum-containing media for 3 weeks and morphology imaged by immunocytochemistry. EF-NC cells were smaller and less mesenchymal in appearance than CV-NC cells (scale bar = 100 µm). (**B**) RT-PCR confirms down regulation of NCSC genes *B3GAT1 (HNK-1)* and *NGFR (p75)* in CV-NC cells after 2 weeks. In contrast, NCSC and EWS-FLI1 target gene expression remained high in EF-NC cells (results for 3 independent experiments are shown). (**C**) Transduced, EGFP+ cells were isolated 6 weeks after transduction and analyzed by western blot. Data from 3 independent experiments show consistent up regulation of BMI-1 and EZH2 and repression of p16 in EF-NC cells. (**D**) After 6 weeks BMI-1, EZH2 and p16 expression in EF-NC cells were equivalent to freshly isolated hNCSC. In contrast, CV-NC cells down regulated polycomb proteins and up regulated p16.

To assess the functional consequences of EWS-FLI1 we monitored 3 different batches of transduced cells for up to 3 months. Whereas CV-NC cells displayed the aforementioned large fibroblastic morphology, EF-NC cells were generally smaller and more NCSC-like in appearance (Fig. 5A). In addition, expression of the NCSC genes *NGFR (p75)* and *B3GAT1 (HNK-1)* was down regulated in CV-NC cells but remained high in EF-NC (Fig. 5B).

Polycomb-mediated gene silencing is an essential feature of stem cell maintenance and is frequently deregulated in cancer [31]. The polycomb proteins BMI-1 and EZH2 are over-expressed by and function as oncogenes in ESFT [7], [23], [32]. Therefore, we evaluated whether the observed maintenance of NCSC features in EF-NC cells was associated with up regulation of these proteins. FACS-sorted, EGFP+ EF-NC and CV-NC were analyzed by western blot after 6 weeks in culture. EF-NC cells reproducibly displayed a marked over-expression of both BMI-1 and EZH2 proteins (Fig. 5C). Interestingly, although the *EZH2* transcript was also significantly induced, transcriptional induction of *BMI-1* was minimal (data not shown) suggesting that post-transcriptional regulation might have mediated BMI-1 protein over-expression. Significantly, up regulation of polycomb proteins was accompanied by silencing of p16 (Fig. 5C) and expression of all 3 proteins in EF-NC cells was equivalent to freshly isolated hNCSC (Fig. 5D). Together these findings corroborate the gene expression data that showed reactivation of a NCSC-like state in EWS-FLI1-transduced hNC-MSC.

### Inhibition of BMI-1 leads to de-repression of p16 and cellular senescence in EWS-FLI1-expressing neural crest cells

**Figure 6:**
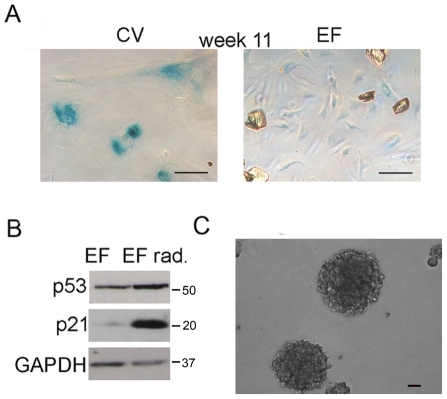
EWS-FLI1 transduced human neural crest cells avoid cellular senescence. (**A**) Senescence-associated beta-galactosidase staining 11 weeks post-transduction shows that CV-NC but not EF-NC cells had undergone senescence (scale bar = 100 µm) (**B**) Functional p53 was confirmed in EF-NC cells by induction of p53 and p21 proteins following gamma irradiation. (**C**) Senescence-resistant EF-NC retain the ability to form neurospheres (scale bar = 100 µm).

**Figure 7:**
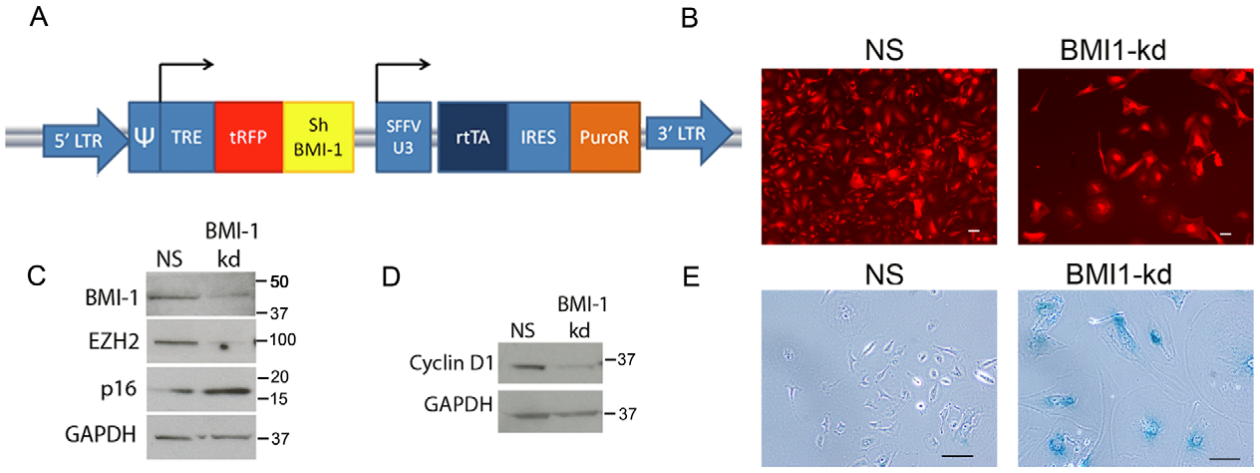
Inhibition of BMI-1 in EF-NC cells restores p16 expression and leads to cellular senescence. (**A**) Inducible BMI-1 knockdown lentiviral vector. (**B**) Red fluorescent protein expression following doxycyline treatment confirmed induction of inert non-silencing (NS) and BMI-1-targeted (BMI1 kd) shRNA in transduced cells (scale bar = 100 µm). (**C**) Western blot analysis confirmed BMI-1 knockdown in BMI1 kd cells accompanied by de-repression of p16 and concomitant down regulation of EZH2. (**D**) BMI-1 knockdown cells also expressed reduced levels of Cyclin D1 compared to control (NS) cells. (**E**) Senescence-associated beta-galactosidase staining 1 week post-doxycyline induction shows that BMI-1 kd cells were senescent (scale bar = 100 µm).

Although both CV-NC and EF-NC cells continued to expand equivalently in 2 of 3 experimental populations, in one experiment CV-NC cells ceased to proliferate after 10 weeks in culture and by 3 months had undergone cellular senescence. In contrast, EF-NC cells continued to proliferate normally (Fig. 6A) and showed no evidence of a secondary loss of p53 function (Fig. 6B). Moreover, these EWS-FLI1+ cells retained the ability to generate neurospheres in anchorage-independent conditions in both defined self-renewal and serum-containing media further supporting maintenance of a stem-like state (Fig. 6C). Silencing of p16 is an initiating event in malignant transformation of primary mammary epithelial cells [33]. To determine if epigenetic silencing of p16 was necessary for continued proliferation and senescence avoidance in EF-NC cells we transduced actively proliferating cells with an inducible BMI-1 shRNA lentiviral construct (Fig. 7A). Exposure of transduced cells to doxycyline led to induction of BMI-1-targeted and inert shRNA sequences as indicated by the appearance of the red fluorescence protein marker (Fig. 7B). Consistent with BMI-1-mediated silencing of p16, knockdown of BMI-1 resulted in de-repression of p16 and, unexpectedly, down regulation of EZH2 (Fig. 7C). Further studies are now required to elucidate the mechanism of this BMI-1/EZH2 interaction and to determine if continued high-level expression of BMI-1 contributes, either directly or indirectly, to maintenance of EZH2 over-expression. Importantly, BMI-1 knockdown cells ceased to proliferate within a few days of doxycycline treatment and cyclin D1 levels decreased (Fig. 7D). One week after initiation of doxycyline the majority of BMI-1 knockdown cells had undergone cellular senescence while control cells continued to proliferate (Fig. 7E).

## Discussion

These studies for the first time demonstrate that, like BM-MSC, neural crest-derived stem cells tolerate EWS-FLI1. Moreover functional studies suggest that the mechanism of oncogene tolerance in these cells is mechanistically linked, at least in part, to up regulation of BMI-1 and epigenetic repression of p16. In addition, gene expression profiling studies reveal a high degree of similarity between ESFT and hNCSC, confirming that activation and maintenance of the NCSC genetic program is an integral feature of ESFT pathogenesis.

Importantly, although tolerance of EWS-FLI1 and up regulation of polycomb proteins was found to be universal in all hNCSC-derived populations, some EWS-FLI1+ cells differentially avoided cellular senescence. These senescence-avoiding cells retained the ability to form neurospheres in non-adherent culture but did not form colonies in soft-agar nor subcutaneous tumors in immune deficient mice (not shown) again demonstrating that like primary human BM-MSC [7], [11], [27], [28], other epigenetic and/or genetic events are necessary to achieve full malignant transformation of human neural crest cells. Technical limitations currently preclude expansion and study of EWS-FLI1 transduced hNCSC at the level of single cells. Therefore, although known to be of neural crest origin, the precise nature of the derivative cells that avoided senescence downstream of EWS-FLI1 activation remains unknown. Nevertheless, gene expression and functional studies support a neuro-mesenchymal stem cell phenotype.

EWS-FLI1 induces cell cycle arrest and death in primary human fibroblasts [4], and initiation of malignant transformation in human BM-MSC [6], [7]. This diversity in functional outcome is reflected in a marked difference in EWS-ETS target genes in disparate cell types [34], [35], [36], [37], a difference which is again highlighted by our current study. Importantly, differences in different cellular contexts are particularly notable when comparing stem cells and established ESFT cell lines. These findings suggest that EWS-FLI1 targets are dynamic and may change during the transition from tumor initiation to tumor maintenance. Continued evolution of transcription factor targets is fundamental to cMYC-induced tumor progression [38] and is no doubt also necessary for successful EWS-FLI1-mediated malignant transformation of primary cells to ESFT.

The mechanisms of EWS-FLI1-mediated gene repression are not yet clearly understood but are likely to be indirect [2]. In normal stem cells, epigenetic repression of developmental genes is largely regulated by polycomb proteins which act together in multi-factorial complexes to modify histones, altering chromatin structure and inhibiting transcriptional activation [39]. Deregulation of polycomb genes is pervasive in human cancer [31] and over-expression of BMI-1 and EZH2 contribute to the tumorigenicity of established ESFT cells [7], [23], [32]. In the current study we have shown that EWS-FLI1 activation leads to a rapid and profound up regulation of BMI-1 protein in hNC-MSC. Our observation that protein induction is more profound than transcriptional induction is consistent with there being a post-transcriptional component to BMI-1 regulation in these cells. Studies in glioma have shown that miRNA128 regulates BMI-1 mRNA and protein expression by directly targeting the 3′-UTR of BMI-1 mRNA [40]. Whether miRNA128 contributes to BMI-1 regulation in ESFT remains to be elucidated. In addition, it has been proposed that in normal stem cells interactive feedback loops exist between PRC1 and PRC2 polycomb group complexes and that these feedback loops lead to altered translation and stability of individual PRC proteins, thereby affecting stem cell function [41]. EWS-FLI1 induces *EZH2* and we have found that knockdown of BMI-1 in neural crest cells also results in down-regulation of EZH2 (Fig. 7C). Further studies are needed to establish the mechanism of BMI-1 upregulation ESFT and its relationship, if any, to *EZH2* induction.

In contrast to *BMI-1*, *EZH2* is a known direct transcriptional target of EWS-FLI1 [32] and is one of 46 genes induced by EWS-FLI1 irrespective of cellular context (Table 1). Moreover, *JARID2*, a recently characterized member of the Jumonji family that complexes with EZH2 to regulate polycomb-mediated gene repression in stem cells [42], was induced by EWS-FLI1 in both hNC-MSC and BM-MSC [7], [26]. Together these findings suggest that abnormal induction of polycomb proteins and subsequent deregulation of polycomb target gene expression might be critical early events in EWS-FLI1-induced transformation.

Successful transformation of primary human cells requires that innate tumor suppressor pathways be repressed. In some experimental models, oncogene-induced transformation has been shown to be dependent on BMI-1-mediated epigenetic repression of the p16/ARF-encoding *CDKN2A* [43], [44]. In addition, epigenetic silencing of p16 is an early event in the transformation of primary human mammary epithelial cells [33], [45]. Our data suggest that epigenetic repression of p16 by BMI-1 may also be an early initiating event in EWS-FLI1-induced malignant transformation. This is in contrast to other studies from our lab which showed that, in established ESFT, BMI-1 contributes to tumorigenicity by means that are largely independent of p16 repression [23]. Thus, as discussed above in reference to EWS-FLI1, disparate molecular mechanisms may contribute to BMI-1's function as an oncogene during tumor initiation and tumor maintenance and we hypothesize that BMI-1 targets change and evolve during ESFT initiation and progression. Further studies are now required to define and compare BMI-1-bound promoters in normal stem cells, their downstream progeny and established tumors.

In summary, we have used a novel model of hESC-derived NCSC to investigate EWS-FLI1 biology and the cellular origins of ESFT. Our findings reveal that ESFT are genetically highly related to NCSC. In addition, neural crest-derived stem cells are permissive for EWS-FLI1 expression and susceptible to oncogene-induced immortalization, at least in part as a result of aberrant up regulation of BMI-1 and epigenetic repression of p16. Together these data support the hypothesis that at least some ESFT might arise from malignant transformation of neural crest-derived stem cells.

## Supporting Information

Table S1 **A.** Significant EWS-FLI1 modulated transcripts. **B.** Overlapping EWS-FLI1 target genes in published datasets.
(XLSX)

Table S2 **A.** NCSC-specific gene signature. **B.** Over-represented gene ontologies among NCSC-specific genes.
(XLSX)

Table S3 **A.** Transcripts specific to NCSC gene signature and significantly altered by EWS-FLI1 in NC-MSC. **B.** Over-represented gene ontologies among NCSC-specific genes that were also significantly altered by EWS-FLI1 in NC-MSC.
(XLSX)

Table S4 **RT-PCR primer sequences.**
(XLSX)
